# *Pseudomonas fluorescens* Cells’ Recovery after Exposure to BAC and DBNPA Biocides

**DOI:** 10.3390/antibiotics11081042

**Published:** 2022-08-02

**Authors:** Ana C. Barros, Luis F. Melo, Ana Pereira

**Affiliations:** 1 LEPABE—Laboratory for Process Engineering, Environment, Biotechnology and Energy, Faculty of Engineering, University of Porto, 4200-465 Porto, Portugal; acbarros@fe.up.pt (A.C.B.); lmelo@fe.up.pt (L.F.M.); 2 ALiCE—Associate Laboratory in Chemical Engineering, Faculty of Engineering, University of Porto, 4200-465 Porto, Portugal

**Keywords:** benzalkonium chloride (BAC), dibromonitrilopropionamide (DBNPA), regrowth, resuscitation, dead cells, viability, persisters, viable but non-culturable cells (VBNC)

## Abstract

A proper assessment of the effects of biocides on bacterial cells is key to the prevention of antimicrobial resistance and the implementation of suitable biocidal programmes. It is particularly relevant regarding the ability of dead-labelled cells to recover their functional processes once the biocide is removed. In the present work, we studied how *Pseudomonas fluorescens* cells previously exposed to different concentrations of BAC (benzalkonium chloride) and DBNPA (2,2-Dibromo-3-nitrilopropionamide) behave upon the restoration of optimum growth conditions. The following indicators were evaluated: culturability, membrane integrity, metabolic activity (resazurin), cellular energy (ATP), and cell structure and morphology (transmission electron microscopy (TEM)). The results demonstrated that cells previously labelled as ‘dead’ recovered to a greater extent in all indicators. Only cells previously exposed to BAC at 160 mg/L (concentration above the MBC) showed significant reductions on all the evaluated indicators. However, the obtained values were much higher than the ‘death’ thresholds found for the autoclaved cells. This suggests that cells exposed to this concentration take more time to rebuild their functional processes. The recovery of DBNPA-treated cells did not seem to be related to the biocide concentration. Finally, a reflection on what kind of cells were able to recover (remaining cells below the detection limit and/or dormant cells) is also presented.

## 1. Introduction

Microbiological control is too often dependent on the use of biocides in a wide range of applications. Well-designed biocidal programmes and proper assessment of the efficacy of disinfection are required to avoid the rise in resistant bacteria, a critical global concern [1]. Understanding the impact of biocides (type, dosage, contact time, etc.) on microorganisms’ viability—are they alive, dead, or dormant?—is key to successfully address this challenge. It was already reported that the continuous exposure of bacteria to stress conditions can lead to irreversible cell death [2]. However, if cells are timely removed from the environment that is causing stress (biocide), they can be repaired and resuscitate [2]. There is a great scientific discussion on the definition of dead cells, as cells can adopt alternative survival mechanisms, and the use of different research methods can lead to different conclusions.

Indeed, bacteria can survive harsh conditions/stress by entering a dormancy state. So far, two distinct dormancy states have been described: persister cells (persisters) and viable but non-culturable (VBNC) cells [3].

Persisters are a subpopulation of non-growing bacteria that can withstand an antibiotic/biocide concentration that, in normal conditions, would kill all the population. Once the antibiotic/biocide is removed, cells continue to grow and become again susceptible to the same antibiotic/biocide. Unlike resistant bacteria that can grow in the presence of antibiotics/biocides and that rely on mutations, persisters are non-genetically modified [3,4,5] cells. VBNC are viable but dormant cells that are not culturable (in conditions known to enable bacterial growth) but that remain metabolically active and retain membrane integrity [2,6]. VBNC cells are in a deeper state of dormancy than persisters [7], and they co-exist and share the same dormant cell phenotype [3,8]. In fact, VBNC cells are persisters, but unlike the latter, VBNC cells take more time to grow on agar plates [8,9]. 

Apart from cells in a dormant state, it is also possible that there are some remaining cells alive but below the detection limit of methods [10,11].

Once the appropriate growth conditions are restored, both dormant cells and remaining cells below the detection limit of the methods, can be reactivated. Such process is named resuscitation when referring to the restoration of dormant cells [12,13,14] and regrowth referring to the growth and division of remaining cells [15,16]. Knowing if cell’s growth is due to resuscitation instead of multiplication of remaining live cells is not straightforward. Many authors claim that the recovery of cells observed in most cases is due to the regrowth of some remaining culturable cells [17,18]. Other studies showed that cells in a dormancy state can also resuscitate [19,20,21,22] when conditions are favourable. Most probably, regrowth and resuscitation co-exist.

There are many cellular processes, parameters, and functions that can be evaluated to access the effect of antimicrobial agents on cells. Too often, those evaluations comprise the use of standard culture methods with well-developed techniques to assess cells’ viability. However, viability output will depend on the method used and on the experimental conditions, as each method uses different parameters based on different reactions and different markers and/or targets. Since there is a great diversity of cell types, it is preferable to assess viability by evaluating multiple parameters in parallel, also taking into account the pros and cons of each method. Furthermore, despite the potential disadvantages of each of the methods per se, when used in combination, they can provide valuable information about the metabolic and physiological states of bacteria.

In a previous work [23], the authors studied in detail the mechanism of action of two widely used biocides: benzalkonium chloride (BAC) and dibromonitrilopropionamide (DBNPA), after exposing *Pseudomonas fluorescens* to different concentrations of such biocides. That work also discussed cells labelled as ‘dead’ based on (i) the assessment of standard indicatorsmethodologies (culturability, membrane integrity, metabolic activity, cellular energy, and transmission electron microscopy (TEM) micrographs); (ii) comparing the results from each indicator to a negative control of cells (without biocidal effect) and a positive control (from autoclaved cells). The combination of information gathered in (i) and (ii) allowed the establishment of a ‘death’ threshold for each methodology. It was found that only the concentrations of 160 mg/L BAC and 35 mg/L of DBNPA (both above the minimum bactericidal concentration (MBC)) matched the death threshold for all indicators. Given these findings, the authors proceeded with the work herein reported to investigate the following questions: (i) can cells previously exposed to BAC and DBNPA recover their functional processes? if so, (ii) to what extent? and (iii) is this recovery dependent on the concentration and/or biocide used? Answers to these questions will also allow an understanding of whether the ‘death’ threshold from autoclaved cells can bring more accuracy to current approaches to study biocides’ antimicrobial efficacy. To address these goals, the biocide was removed from the bacterial suspension, and *P. fluorescens* cells were supplied with a fresh nutrient medium and incubated overnight at optimal growth conditions (agitation and temperature). Then, the same parameters analysed by Barros et al. [23] were evaluated, namely culturability, viability (PI uptake), metabolic activity (resazurin), cellular energy (ATP), and structural and morphological changes (TEM imaging).

## 2. Results and Discussion

To understand if *Pseudomonas fluorescens* cells previously exposed to different concentrations of BAC and DBNPA can recover, cells were supplied with a fresh nutrient medium and allowed to grow overnight, defined as an ‘overnight step’. Recovery was evaluated in terms of culturability, membrane integrity, metabolic activity, cell energy, and cell structure and morphology.

### 2.1. Culturability

**Figure 1:**
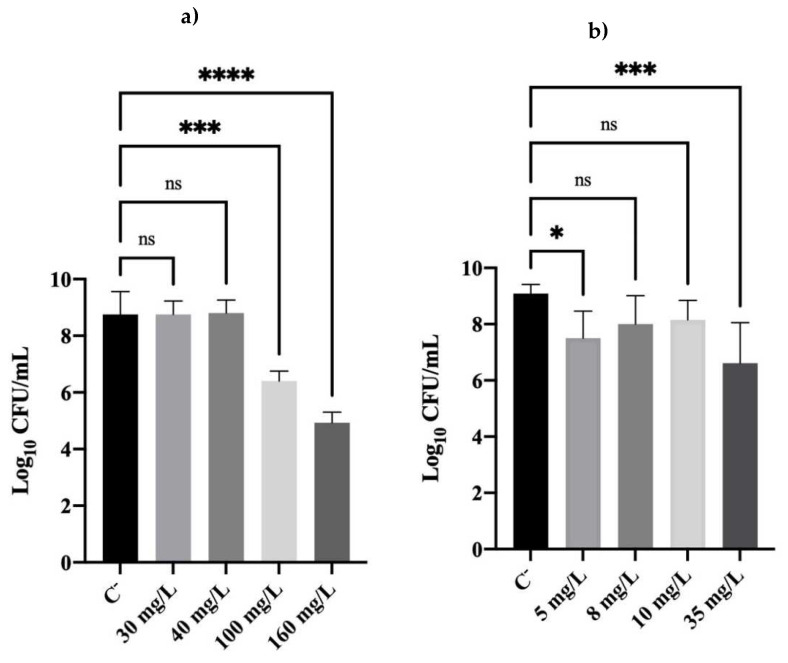
Logarithm of the CFUs/mL of *Pseudomonas fluorescens* cells after an overnight step in a nutrient medium. Cells had been previously exposed to different concentrations of (**a**) BAC and (**b**) DBNPA for 30 min. Controls were performed with untreated cells (C^−^). The means ± standard deviation of three independent experiments are presented. ‘ns’ indicates not significant (*p* > 0.05), whereas the asterisks indicate statistical significance (* *p* < 0.05; *** *p* < 0.001; and **** *p* < 0.0001), using Tukey’s multiple comparison test.

The culturability of cells after an overnight step was evaluated, and the results are depicted in Figure 1. 

Figure 1a shows that *P. fluorescens* cells previously exposed to 30 and 40 mg/L of BAC reached the same number of culturable cells ~9 log_10_ CFU/mL (*p* > 0.05) as C^−^ (untreated cells) after regrowth. Lower log_10_ CFU/mL were observed for the MBC (100 mg/L) and for 160 mg/L, which reached, respectively, 6.4 and 5 logs. These reductions were significantly different than that in C^−^. 

Concerning DBNPA-exposed cells, Figure 1b shows that cells were able to regrow to a greater extent (between 7 and 8 logs) no matter the biocidal concentration. There was a statistical difference between the control and 5 mg/L concentration (*p* < 0.05) and 35 mg/L (*p* < 0.001) but not for the intermediate concentrations. This is probably related to the fact that CFU/mL values at the DBNPA concentrations of 5, 8, and 10 mg/L were not significantly different, and the overall standard deviations of the three independent trials affected the overall statistical analysis of the 5 mg/L concentration.

The overall culturability results show that cells were able to recover from BAC and DBNPA exposure, after an overnight assay at optimal growth conditions, for all tested concentrations. The definition of MBC is the lowest biocidal concentration able to kill 99.9% of the initial bacterial population [24]. Therefore, when cells were first exposed to the MBC and higher concentrations [23], no CFU/mL counts were observed. This means that, for both biocides, cells were able to significantly regrow when stress conditions (biocide effect) were removed, and nutritional requirements were met.

### 2.2. Membrane Integrity

**Figure 2:**
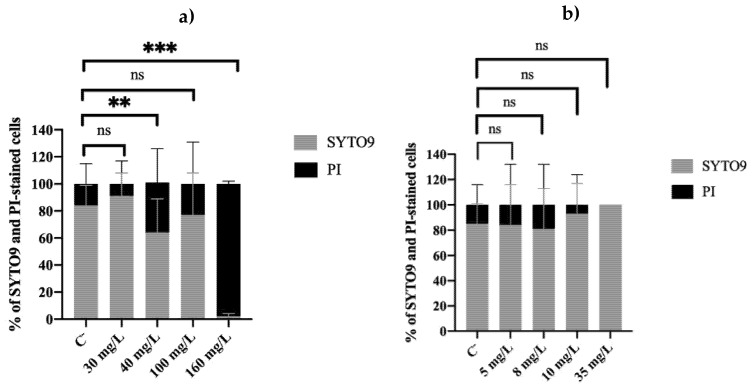
Percentage of *Pseudomonas fluorescens* cells stained with SYTO9 (green cells, light grey) and propidium iodide (red cells, black) after an overnight step in a nutrient medium, resulting from previous exposure to different concentrations of (**a**) BAC and (**b**) DBNPA for 30 min. Controls were performed with untreated cells (C^−^). The means ± SD of three independent experiments are presented. ‘ns’ indicates not significant (*p* > 0.05), whereas the asterisks indicate statistical significance (** *p* < 0.01 and *** *p* < 0.001), using Tukey’s multiple comparison test.

Membrane integrity of biocide-exposed cells was also evaluated (Figure 2).

The work of Barros et al. [23] discussed that BAC, due to its cationic nature, strongly interacts with bacterial cell membranes, inducing small membrane pores that allow PI entrance into the cells. The authors demonstrated that only 30 mg/L of BAC can lead to 100% of PI uptake, even though *P. fluorescens* remained culturable for concentrations below the MBC. On the other hand, in the present study, Figure 2a shows low PI uptake percentages after regrowth, meaning that PI could not cross bacterial membranes. This seems to agree with Davey [25], who showed that PI can enter microbial cells immediately after exposure to stress, but after a short period of time, the cell’s membrane can be repaired in such a way that PI can no longer enter. The only exception is the bacterial cells exposed to 160 mg/L of BAC (*p* < 0.001), which showed PI uptake percentages (98%) indicating the existence of significant membrane injuries after regrowth. However, the comparison of membrane integrity with culturability raises some questions, as the cells were able to grow in a solid medium even when they had damaged membranes. These discrepancies can possibly be explained by the fact that PI is able to penetrate viable cells with high membrane potential [26,27], which can lead to a misinterpretation of the results. Additionally, since 160 mg/L is above the MBC, it caused higher damage to the cells [23], making it more difficult for cells to recover their membrane integrity. 

Regarding DBNPA-exposed cells, Figure 2b shows that the PI uptake percentages for the different concentrations were similar to those of C^−^ (ranging between 0% and 19%). These findings are in accordance with the PI uptake values observed when cells were first exposed to DBNPA. Barros et al. [23] concluded that the mechanism of action of this biocide interferes with the components inside the cell, leading to intracellular components leaking, without significantly affecting (for 30 min contact time) the cell’s membrane. The opposite effect observed for BAC may be related to its lytic mechanism of action [28,29], which is known to interfere with the cell wall. 

### 2.3. Metabolic Activity

**Figure 3:**
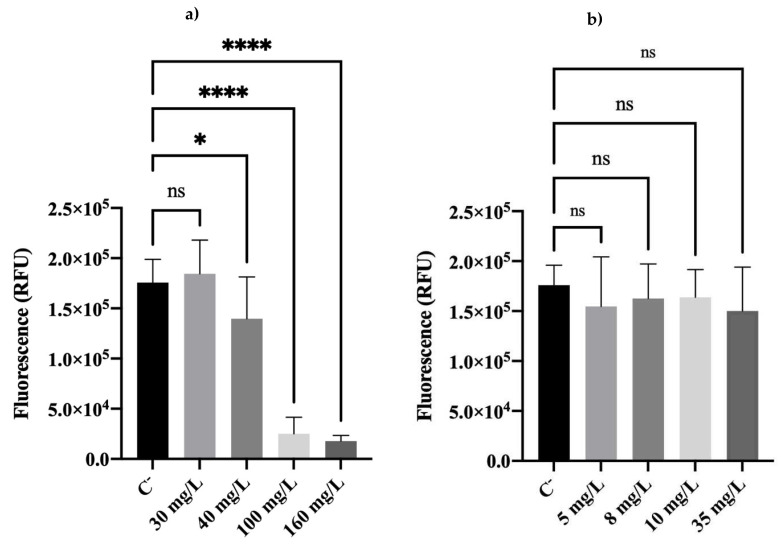
Fluorescence of *Pseudomonas fluorescens* cells stained with resazurin after an overnight step in a nutrient medium, resulting from previous exposure to different concentrations of (**a**) BAC and (**b**) DBNPA for 30 min. Controls were performed with untreated cells (C^−^). The means ± SD of three independent experiments are presented. ‘ns’ indicates not significant (*p* > 0.05), whereas the asterisks indicate statistical significance (* *p* < 0.05 and **** *p* < 0.0001), using Tukey’s multiple comparison test. RFU: relative fluorescence units.

To evaluate if the cell’s viability was recovered after the regrowth period, the metabolic activity of biocide-exposed cells was assessed using the resazurin dye assay (Figure 3). Cells previously exposed to BAC (Figure 3a) at 40, 100, and 160 mg/L showed significantly lower fluorescence (metabolic activity) values than C^−^. Conversely, no significant differences were observed between DBNPA-exposed cells and C^−^ (Figure 3b).

In the work of Barros et al. [23], when assessing the effect of biocides on *P. fluorescens* cells’ metabolic activity, the authors observed that (i) the threshold of fluorescence intensity observed for dead cells (autoclaved cells) was 1.1 × 10^4^ RFUs, and (ii) only cells exposed to 160 mg/L (BAC) and 35 mg/L (DBNPA) reached the death threshold. 

In the present study, only the cells exposed to 160 mg/L of BAC showed a fluorescence intensity value (1.8 × 10^4^) matching the death threshold. All the other concentrations of both biocides showed values much higher, suggesting that cells recovered their metabolic activity.

### 2.4. Cellular Energy

**Figure 4:**
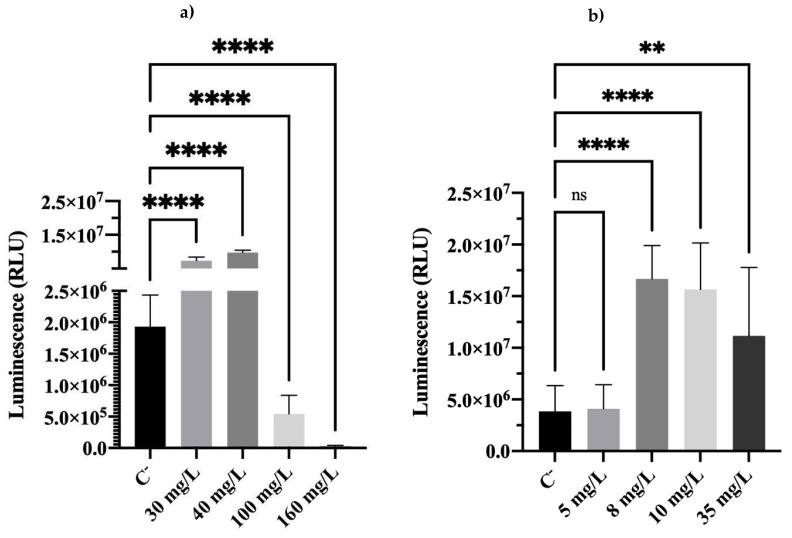
Luminescence of *Pseudomonas fluorescens* cells after an overnight step in a nutrient medium, resulting from previously exposure to different concentrations of (**a**) BAC and (**b**) DBNPA for 30 min. Controls were performed with untreated cells (C^−^). The means ± SD of three independent experiments are presented. “ns” indicates not significant (*p* > 0.05), whereas the asterisks indicate statistical significance (** *p* < 0.01 and **** *p* < 0.0001), using Tukey’s multiple comparisons test. (RLU: Relative Light Units).

Cellular energy (in terms of ATP) after the overnight growth step was also evaluated (Figure 4) to obtain additional information on the cell’s viability, as adenosine triphosphate (ATP) is a key energy carrier in the cells.

Figure 4a shows that cells previously exposed to BAC, regardless of the tested concentration, had different ATP contents, compared with C^−^. Similar results were found for DBNPA (Figure 4b), which, except for the 5 mg/L sample, showed statistically significant different luminescence values for all tested concentrations. It is interesting to note that some samples showed significantly higher ATP levels than C^−^ (for both biocides): BAC at 30 and 40 mg/L (Figure 4a) and DBNPA at 8, 10, and 35 mg/L (Figure 4b). Akhova and Tkachenko [30] also observed an increase in ATP content after exposing *Escherichia coli* cells to antibiotics. The authors concluded that the ATP increase was not related to an increase in bacterial respiration but instead to the inhibition of energy-consuming processes that lead to the accumulation of ATP inside the cells [30]. This also seems to agree with former works in which energy accumulation inside stressed cells was found to help cells recover from injuries [18,31,32]. Another hypothesis might be related to the findings that high ATP levels can be indicative of the presence of VBNC cells [32,33,34,35].

Furthermore, in our previous study [23] it was reported that (i) the death threshold (via analysis of autoclaved cells) for ATP content was 7.9 × 10^3^ RLUs; (ii) the death threshold for ATP was reached when cells were exposed to 40 mg/L of BAC and 35 mg/L of DBNPA. It can be seen from Figure 4 that, after the overnight step, ATP levels were substantially higher than the ones found immediately after biocide exposure. Therefore, in most cases, cells that were previously labelled as ‘dead’ according to the ATP indicator regained viability to a greater extent. Only the cells previously exposed to BAC at 160 mg/L showed an ATP content that was below the death threshold. This result agrees with membrane integrity and metabolic activity data. Furthermore, the lytic nature [28,29] of this biocide can explain the low amounts of ATP found upon biocide exposure [23], and it is probably the reason why cells have a lower ATP content after recovery. The holes caused by BAC action led to the release of high amounts of ATP, and therefore, it may have been more difficult for cells exposed to such high concentrations to restore ATP levels during recovery.

### 2.5. Cell Structure and Morphology

**Figure 5:**
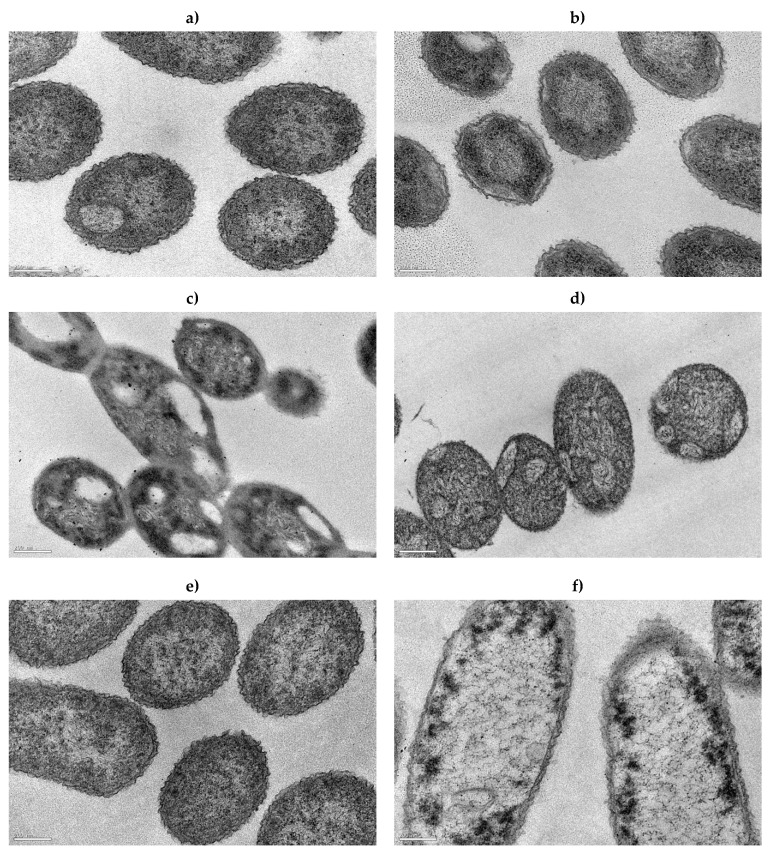
Transmission electron micrographs (80,000×) of *P. fluorescens* cells after biocide exposure (**a**,**c**,**e**) and after an overnight step (**b**,**d**,**f**) of (**a**,**b**) untreated C^−^; (**c**,**d**) previously exposed for 30 min to BAC MBC (100 mg/L); and (**e**,**f**) previously exposed to DBNPA MBC (10 mg/L). Scale bar = 200 nm.

The cells’ structure and morphology are depicted in Figure 5. Figure 5c,e show the effect of exposure to the MBCs of the tested biocides (BAC and DBNPA), whereas Figure 5d,f are related to the cell’s recovery.

TEM micrographs of untreated (C^−^) *P. fluorescens* cells (Figure 5b) show intact cell membranes and a high and uniform electron density. Comparing Figure 5a,b, it is also possible to see that the structure and morphology of these cells (C^−^) remained similar after recovery.

On the other hand, as previously reported by Barros et al. [23], the exposure of cells to biocides significantly affected the cell structure: BAC disrupted the cell wall and led to the release of intracellular contents (Figure 5c). However, Figure 5d shows that those damages were less notorious after the overnight step. Despite the presence of vacuoles as before (Figure 5c), there was an increase in electron density homogeneity, suggesting that cells were able to repair.

As described in Barros et al. [23], and illustrated in Figure 5e, the DBNPA-exposed cells showed intact membrane envelopes and intracellular content leakage. After recovery, cells seemed to keep a similar morphology (Figure 5f), but there was an apparent increase in the leakage of intracellular content when compared with the cells immediately after biocide exposure. This characteristic could be an indication of cell death; however, from the results discussed so far, it is more likely that this is related to the existence of VBNC cells [3]. VBNC cells can have a cytosol with low density, yet still enough to maintain the basic metabolic activities of cells [7].

### 2.6. To What Extent Can Dead-Labelled Cells Become Alive When a Friendly Environment Is Met?

Comparing all the results from BAC-exposed cells, and regardless of the concentration, cells became culturable (Figure 1) and viable (Figure 2, Figure 3 and Figure 4) after the re-establishment of the optimum growth conditions. Cells previously exposed to higher (equal or above the MBC) concentrations of BAC, i.e., 100 and 160 mg/L, showed lower values of culturability, metabolic activity, and cellular energy. This suggests that higher BAC concentrations can slow down the re-establishment of cellular functions. This effect is particularly noticeable in the membrane integrity results, as cells exposed to different concentrations recovered their membrane integrity (low PI uptake) in comparison with the results after biocide exposure [23], which showed PI percentage uptakes compatible with high membrane damage. As discussed in Section 2.2, even cells previously exposed to 160 mg/L of BAC (Figure 2a), which remained with injured membrane cells (high PI uptakes), recovered the ability to grow on solid media (Figure 2a). Additionally, TEM results corroborate the re-establishment of cell structure and morphology (Figure 5d).

Similarly, cells previously exposed to DBNPA, no matter the concentration, recovered their functional processes in all the evaluated indicators. However, TEM micrographs (Figure 5f) suggested that cells did not fully recover, as an apparent leakage of cellular content was observed. As argued, this is most likely related to the fact that cells retrieve from a VBNC state. This hypothesis is reinforced by the luminescence results, which can also be indicative of the presence of VBNC cells, as higher ATP levels have been attributed to VBNC cells [36,37,38]. Nonetheless, as discussed by Barros et al. [23], the calculation and interpretation of the VBNC state from cells exposed to BAC and DBNPA, using PI as a viability indicator, are misleading, as BAC and DBNPA interact very differently with the cell wall. This fact does not allow a proper assessment of the number of VBNC cells.

Finally, contrary to BAC, the recovery of cells exposed to DBNPA upon the overnight step did not seem to be concentration-dependent, since the recovery results were similar regardless of the concentration of DBNPA. This distinct behaviour might be related to the mechanism of action of each biocide, which is very different for BAC and DBNPA, according to Barros et al. [23]. Additionally, the overall results suggest that BAC was more effective in preventing bacterial recovery after cells were exposed to biocidal concentrations equal to (100 mg/L) or above (160 mg/L) the MBC concentration. A significant reduction in all tested indicators was observed in comparison with the untreated cells.

Apart from the biocides’ mechanism of action, the overnight step conditions [39,40] (time, temperature, and nutritional medium) also played an important role on cells’ recovery time and extent. Taking this into account, the overnight step conditions used in this work were chosen to match the optimal growth conditions of normal *P. fluorescens* cells.

### 2.7. What Kind of Cells Recovered? 

Given the former results, it is worthy to reflect on what kind of cells were able to recover their functional processes. Two possibilities might be considered: (i) cells recovered from a dormancy state (e.g., persister (shallow dormancy state) and VBNC (deeper dormancy state cells)) via a process named resuscitation; and/or (ii) some remaining cells that were under the limit of detection of the methods and that grew and multiplied, through a process named regrowth [15,16]. In the present work, it seemed that the type and concentration of biocide that cells have been previously exposed to influenced whether the remaining live cells regrew and/or the dormant ones resuscitated. In the former work, Barros et al. [23] found that, after *P. fluorescens* cells were exposed to BAC and DBNPA concentrations below the MBC, a high concentration of remaining cells (culturable and viable) was still observed. Thus, the high logs of CFU/mL observed in Figure 1 (for concentrations below the MBC) are most likely due to those remaining cells that, upon nutrition replenishment, regrew. However, when considering, for example, the cells previously exposed to BAC at the MBC and higher concentrations, it is unlikely that the cells’ recovery was only due to the remaining culturable (regrowth) cells, as the culturability detection limit was 1.7 log. If only these 1.7 log_10_ CFU/mL were responsible for the growth observed, the culturability results would be expectedly lower than C^−^. However, 6.4 log_10_ CFU/mL have still been able to recover from the BAC MBC concentration, suggesting that a fraction of the dormant cells were able to resuscitate and regain culturability. Indeed, in the work of Fernandes et al. [41], it was reported that *P. fluorescens* persister cells were also able to recover after biocide (BAC) removal and nutrient re-establishment. Although in that paper, persisters’ formation resulted from the BAC effects on 48 h old biofilm cells, and the contact time and concentration used were different from those of the present study, a similar trend was observed. 

This is even more evident in the DBNPA results, as the cells previously exposed to the highest concentration (10 mg/L) and previously labelled as ‘dead’ according to all indicators [23] and to the ‘death’ threshold, were able to resuscitate to a greater extent in all the evaluated parameters. Resuscitation might occur from VBNC or persister cells; however, in the case of DBNPA, as discussed in the previous section, several indicators point out to the existence and recovery of VBNC cells.

## 3. Materials and Methods

### 3.1. Biocides

*Two* biocides were used in this study: benzalkonium chloride (BAC, Sigma-Aldrich, Søborg, Denmark) and 2,2-Dibromo-3-nitrilopropionamide (DBNPA, provided by Enkrott^®^, S.A, Sintra, Portugal). Biocide solutions were aseptically prepared in ultrapure water (UPW) and stored at 4 °C.

### 3.2. Microorganism and Culturing Conditions

*Pseudomonas fluorescens* isolated from a drinking water distribution system and identified via 16S rRNA gene sequencing [42] was used in this study. Bacterial cells were grown overnight in a batch culture in a nutrient medium comprising 5 g/L glucose (Merck, Darmstadt, Germany), 2.5 g/L peptone (Merck, Darmstadt, Germany), and 1.25 g/L yeast extract (Merck, Darmstadt, Germany), in a 0.02 M phosphate buffer with pH 7 (KH_2_PO_4_; Na_2_HPO_4_—Chem-Lab NV, Zedelgem, Belgium) at 30 °C and under 120 rpm.

### 3.3. Recovery Ability after Biocide Exposure

Based on a previous study [23], the cells were exposed to biocides (BAC and DBNPA) at the minimum bactericidal concentration (MBC), two concentrations below the MBC and one concentration above the MBC. For BAC, the following concentrations were tested: 30, 40, 100 (MBC), and 160 mg/L. For DBNPA, the studied concentrations were 5, 8, 10 (MBC), and 35 mg/L. Additionally, controls with untreated cells (C^−^) were performed. Therefore, bacterial cells from the biocide exposure assays [23] were centrifuged (4000 rpm, 5 min) and resuspended in the nutrient media. The cells were placed in 25 mL flasks and left overnight (16–18 h) under agitation (120 rpm) at 30 °C. This recovery time was chosen since it is the time that *P. fluorescens* cells need to reach the exponential growth phase. Afterwards, the cells were centrifuged (4000 rpm, 5 min), washed with saline solution, and centrifuged again (4000 rpm, 5 min). The pellets were resuspended in saline solution (0.85 % *v*/*v*) and analysed in terms of culturability, membrane integrity, metabolic activity, cellular energy, and structural and morphological changes. These methods are briefly described in this section.

For culturability analysis, the drop plate method was used [43]. The suspensions were serially diluted and plated on plate count agar (PCA- Merck, Darmstadt, Germany), followed by incubation overnight at 30ºC. The number of colonies was determined, and the results are presented as the logarithm of the colony-forming units per millilitre (log_10_ CFU/mL). Three independent experiments were performed for each condition.

The membrane integrity was analysed using a LIVE/DEAD^®^ Baclight^TM^ kit (Invitrogen/Molecular Probes, Eugene, OR, USA). The nucleic acids SYTO9^TM^ and propidium iodide (PI) were used to stain intact and damaged membranes, respectively. In brief, bacterial cells were stained in the dark with the two dyes and filtered through a 0.2 μm Nucleopore^®^ (Whatman, Middlesex, UK) black polycarbonate membrane. Afterwards, the membranes were mounted on a slide with immersion oil and observed using a LEICA DMLB2 microscope (LEICA Microsystems Ltd., Wetzlar, Germany). At least 15 images were recorded for each sample, and the data are presented as SYTO9 and PI uptake percentages. Three independent experiments were performed for each condition.

The metabolic activity was evaluated using a resazurin assay. Cell suspensions were placed in contact with resazurin (final concentration 20 μM) in 96-well microtiter plates. The plates were protected from light, and the fluorescence (in relative fluorescence units (RFU)) was recorded (λ_excitation_ = 570 nm and λ_emission_ = 590 nm) after 24 h on a FLUOstar^®^ Omega microtiter plate reader (BMG Labtech, Ortenberg, Germany). Three independent experiments were performed for each condition. 

For the cellular energy (ATP) determination, a BacTiter-Glo^TM^ Microbial Cell Viability Assay (Promega, Madison, WI, USA) was used. After mixing the BacTiter-Glo^TM^ reagent with bacterial cells in 96-well white microtiter plates, the luminescence (in relative luminescence units (RLU)) was assessed using a FLUOstar^®^ Omega microtiter plate reader (BMG Labtech, Ortenberg, Germany). Three independent experiments were performed for each condition.

Structural and morphological changes were evaluated using transmission electron microscopy (TEM), with the protocol described in Barros et al. [23]. Images were taken using a JEOL JEM 1400 microscope (JEOL, Tokyo, Japan).

Further details on the methods can be found in the previous work by Barros et al. [23].

### 3.4. Statistical Analysis

Statistical analysis was performed using GraphPad Prism version 9.0.1 for macOS software (GraphPad Software, San Diego, CA, USA). Data were analysed using one-way ANOVA with Tukey’s multiple comparison test. All samples were compared with C^−^. A confidence level of 95% (*p* < 0.05) was used as statistical significance. All the data are expressed as means ± standard deviation (SD) of three independent experiments.

## 4. Conclusions

The present work, discusses to what extent *P. fluorescens* cells previously exposed to biocides (BAC and DBNPA) can recover their functional behaviour and morphology upon biocide removal and the re-establishment of optimum growth conditions. To this end, and as a complement to the indicators used (culturability, membrane integrity, metabolic activity, cellular energy, and cells’ structure and morphology), the death thresholds from autoclaved cells reported in our previous work [23] were used as guidance for a more accurate interpretation. The results showed that cells previously exposed to a wide range of biocidal concentrations of BAC and DBNPA regained culturability, restored the integrity of the cell wall, and recovered metabolic activity and cellular energy after recovery. These results were particularly surprising for the MBC concentrations of both biocides (100 mg/L of BAC and 10 mg/L of DBNPA), as they were previously labelled as ‘dead’ [23] in comparison to the ‘dead threshold’ given by the autoclaved cells. Furthermore, the biocides’ mechanism of action and the prior concentrations that cells were previously exposed to seemed to affect the regrowth/resuscitation extent. 

Additionally, care must be taken when using a single method to assess cell viability, since the existence of persisters and VBNC cells can be neglected by some techniques.

## Data Availability

Not applicable.
